# Improved multiplex PCR primers for rapid identification of coagulase-negative staphylococci

**DOI:** 10.1007/s00203-017-1415-9

**Published:** 2017-08-09

**Authors:** Jonguk Kim, Jisoo Hong, Jeong-A Lim, Sunggi Heu, Eunjung Roh

**Affiliations:** 1 Microbial Safety Team, National Institute of Agricultural Sciences, Rural Development Administration, Wanju, 55365 Republic of Korea; 2 Crop Cultivation and Environment Research Division, National Institute of Crop Science, Rural Development Administration, Suwon, 16613 Republic of Korea

**Keywords:** Coagulase-negative staphylococci, Species-specific PCR, *sodA*, Antimicrobial resistance, Virulence factor

## Abstract

Coagulase-negative staphylococci (CNS) are opportunistic pathogens that are currently emerging as causative agents of human disease. Though CNS are widespread in the clinic and food, their precise identification at species level is important. Here, using 16S rRNA sequencing, 55 staphylococcal isolates were identified as *S. capitis, S. caprae, S. epidermidis, S. haemolyticus, S. pasteuri, S. saprophyticus, S. warneri,* and *S. xylosus.* Although 16S rRNA sequencing is universally accepted as a standard for bacterial identification, the method did not effectively discriminate closely related species, and additional DNA sequencing was required. The divergence of the *sodA* gene sequence is higher than that of 16S rRNA. To devise a rapid and accurate identification method, *sodA*-specific primers were designed to demonstrate that species-specific multiplex polymerase chain reaction (PCR) can be used for the identification of CNS species. The accuracy of this method was higher than that of phenotypic identification; the method is simple and less time-consuming than 16S rRNA sequencing. Of the 55 CNS isolates, 92.72% were resistant to at least one antibiotic, and 60% were resistant to three or more antibiotics. CNS isolates produced diverse virulence-associated enzymes, including hemolysin (produced by 69.09% of the isolates), protease (65.45%), lipase (54.54%), lecithinase (36.36%), and DNase (29.09%); all isolates could form a biofilm. Because of the increasing pathogenic significance of CNS, the efficient multiplex PCR detection method developed in this study may contribute to studies for human health.

**Electronic supplementary material:**

The online version of this article (doi:10.1007/s00203-017-1415-9) contains supplementary material, which is available to authorized users.

## Introduction

Staphylococci includes coagulase-positive staphylococci (CPS), almost exclusively represented by *Staphylococcus aureus*, and coagulase-negative staphylococci (CNS) (Becker et al. 2004). To date, the *Staphylococcus* genus comprises 49 species and 26 subspecies (Han et al. 2015). Most studies on staphylococcal pathogenicity have focused on *S. aureus*, and little attention has been paid to CNS (Chajecka-Wierzchowska et al. 2015). CNS have been considered as nonpathogenic; however, they were recently isolated from patients with a weakened immune system and identified as the causative agents of infections caused by contaminated medical equipment and food (Piette and Verschraegen 2009). Most CNS infections occur as a consequence of long-term usage of indwelling medical devices, such as central venous catheters, artificial heart valves, and pace-makers (Chu et al. 2008). Because CNS are found on normal human and animal skin, fresh vegetables can be contaminated by hand contact during harvest or distribution. The foodstuff surface can serve as a vehicle for the transmission of pathogenic bacteria capable of causing disease in humans (Maistro et al. 2012; Marino et al. 2011). CNS infections are difficult to control because these bacteria produce biofilms on the surfaces of foreign materials and are resistant to multiple antibiotics (John and Harvin 2007). Biofilm formation increases their antibiotic resistance by about 1000-fold over that of planktonic bacteria (Donlan 2002). In addition to biofilms, CNS species produce other diverse virulence factors and enzymes, such as hemolysin, lipase, lecithinase, DNase, and protease (Kot et al. 2013). Alarmingly, a significant increase of multidrug-resistant CNS infections is observed (Koksal et al. 2009). Because of the increasing pathogenic significance of CNS, rapid and accurate identification methods are required for the assessment of the pathogenic potential of individual CNS species and for the development of species-specific management strategies.

Various CNS identification methods exist, both phenotypic and genotypic. Many different types of manual and automated tests have been developed for the identification of CNS species based on their phenotypic characteristics. These include the API Staph test, Staph-Zym test, and BD Phoenix Automated Microbiology System (Aldea-Mansilla 2006; Cirkovic et al. 2008); however, the accuracy of these methods is low, 50–70% (Koop et al. 2012). Recently, several attempts have been made to identify CNS species using matrix-assisted laser desorption ionization time-of-flight mass spectrometry (MALDI-TOF MS) based on different protein expression profiles (Dupont et al. 2010). However, large variation in the sample treatment methods makes it difficult to identify pathogens in mixed cultures using this procedure. Furthermore, the use of this method is associated with a high initial acquisition cost, which limits its implementation in many laboratories (Tomazi et al. 2014). By comparison, genotypic identification methods based on DNA sequencing are more accurate than the phenotypic tests mentioned above (Bergeron et al. 2011). Amplified fragment length polymorphism fingerprinting (AFLP) (Taponen et al. 2006) is relatively discriminative, but is expensive and labor intensive. Whole-genome DNA–DNA hybridization analysis (Svec et al. 2004) has also been used for the identification of *Staphylococcus* species. These two methods are not suitable for routine use, and their major disadvantages include sample manipulation after the polymerase chain reaction (PCR) step and the requirement for gene probes, whose preparation may be time-consuming.

Partial 16S rRNA sequencing is widely accepted as a standard method for bacterial identification (Becker et al. 2004). However, in some cases, the method is unable to discriminate between phylogenetically close species (Ghebremedhin et al. 2008). Species identification methods have therefore been developed based on sequencing of other housekeeping genes, including *hsp60* (encoding heat shock protein 60) (Goh et al. 1997), *gap* (glyceraldehyde-3-phosphate dehydrogenase) (Yugueros et al. 2000), *sodA* (superoxide dismutase A) (Poyart et al. 2001), *tuf* (elongation factor Tu) (Martineau et al. 2001), *rpoB* (β subunit of RNA polymerase) (Drancourt and Raoult 2002), and *dnaJ* (chaperone DnaJ) (Shah et al. 2007). Sequence variation within the *sodA* gene has been widely exploited for the genotypic identification of CNS species (Sivadon et al. 2005). The divergence of the staphylococcal *sodA* gene is higher than that of 16S rRNA (Poyart et al. 2001); thus this gene has been used as a target in staphylococcal species identification (Iwase et al. 2007). However, these studies require additional processes after the PCR step, such as DNA sequencing and homology comparisons.

In the current study, new primer sets targeting *sodA* were employed in species-specific multiplex PCR for rapid and accurate identification of CNS species. Antimicrobial resistance and virulence factors produced by the CNS isolates were also evaluated to investigate their pathogenicity.

## Materials and methods

### Isolation of staphylococci

Staphylococcal isolates (*n* = 55) used in this study were collected from leaf vegetables, including lettuce, perilla leaf, and chicory, from local markets in South Korea. Eight American Type Culture Collection (ATCC) strains were used as reference strains (Table 1). For each sample, 10 g of leaves was added to 90 mL of buffered peptone water (Difco, Sparks, MD, USA), homogenized for 60 s in a stomacher(Interscience, Saint Nom, France) and incubated for 24 h. Samples were cultured on selective Baird–Parker agar (Difco). Staphylococci were randomly isolated based on colony morphology and grown on Tryptic Soy Agar (TSA; Difco) medium at 37 °C.

**Table 1:** Reference strains used in this study

Species	Strain
*S. capitis* subsp. *capitis*	ATCC 27840
*S. caprae*	ATCC 35538
*S. epidermidis*	ATCC 14990
*S. haemolyticus*	ATCC 29970
*S. pasteuri*	ATCC 51129
*S. saprophyticus* subsp. *saprophyticus*	ATCC 15305
*S. warneri*	ATCC 29885
*S. xylosus*	ATCC 29971

*ATCC* American Type Culture Collection

### Identification

The phenotypic identification of staphylococci was performed based on colony morphology on Baird–Parker agar, Gram staining, and the API Staph test (BioMerieux, Marcy-l’Étoile, France). The API Staph test was performed according to the manufacturer’s instructions, and the results were interpreted using apiwebTM (https://apiweb.biomerieux.com). The genotypic identification of staphylococci involved sequence analysis of 16S rRNA. The 16S rRNA gene from 55 CNS isolates was PCR-amplified using the universal primers 518F (5′-CCAGCAGCCGCGGTAATACG-3′) and 800R (5′-TACCAGGGTATCTAATCC-3′).

### DNA extraction

Bacterial cells from 3 mL overnight cultures were harvested by centrifugation at 3000×*g*. Harvested cells were resuspended in 0.1 mL of TE buffer (10 mM Tris–HCl and 1 mM EDTA, pH 8.0) containing 5 μL of proteinase K (20 mg/mL) and 5 μL of lysostaphin (10 mg/mL), and were incubated for 60 min at 37 °C. Subsequently, 0.1 mL 10 mM Tris–HCl (pH 8.0) and 60 μL of lysozyme (10 mg/mL) were added, followed by incubation at 37 °C for 1 h. After pre-treatment, the DNA was extracted using a G-spin Genomic DNA Extraction Kit (Intron Biotechnology, Kyungki-Do, South Korea).

### Species-specific PCR

To design species-specific PCR primers, the sequence of the *sodA* gene from each CNS species was downloaded from GenBank (National Center for Biotechnology Information, Bethesda, MD, USA) and analyzed. Sequences from the following organisms were used: *S. capitis* (AJ343896), *S. caprae* (AJ343898), *S. haemolyticus* (AJ343910), *S. pasteuri* (AJ343920), *S. saprophyticus* (AJ343924), *S. warneri* (AJ343932), and *S. xylosus* (AJ343933). After comparing the *sodA* sequences from the seven species, specific regions where the sequences diverged were chosen as candidate primer sequences. In the case of *S. epidermidis*, there was no specific sequence in *sodA* to discriminate *S. epidermidis* from other species; therefore, primers targeting the *gseA* gene (encoding endopeptidase A) were used instead (Byrne et al. 2007). After confirming the specificity of these candidates via PCR with genomic DNA from each species as a template, species-specific PCR primer sets were selected. The primer sequences used in this study are shown in Table 2. Seven primer sets targeting the *sodA* gene were used to amplify targeted fragments from the seven CNS species (*S. capitis, S. caprae, S. haemolyticus, S. pasteuri, S. saprophyticus, S. warneri,* and *S. xylosus*). Purified genomic DNAs from the 55 staphylococcal isolates and reference strains were used in amplification reactions with these primer sets.

**Table 2:** PCR primers used in this study

Group	Target microorganism	Target gene	Primer	Sequence (5′–3′)	Amplicon size (bp)	Annealing temperature (°C)	References
1	*S. xylosus*	*sodA*	SX297F	GCAAATCTAGACAGTGTTCCAGAAAAT	297	63	In this study
			SX297R	CTTCTGAGTTTGGAGTTAAT			
	*S. pasteuri*	*sodA*	PA237F	GCTAATTTAGACAGTGTACCTTCTG	237	61	In this study
			PA237R	GCCCGTTATTTACTACTAACCA			
	*S. warneri*	*sodA*	SW110F	GTAACAAAATTAAATGCAGCTG	110	57	In this study
			SW110R	TCTTACTGCAGTTTGAATATCAGA			
	*S. haemolyticus*	*sodA*	HA54F	AAACAAACTATGGAAATCCATCATG	54	58	In this study
			HA54R	ATTTGGTAACATACGTGTTGTG			
2	*S. caprae*	*sodA*	CR252F	AATTTAGATAGCGTACCTTTG	252	58	In this study
			CR252R	AGTTACGATTTCTAATTGACCGTT			
	*S. epidermidis*	*gseA*	Epi F	GGCAAATTTGTGGGTCAAGA	194	65	(Byrne et al. 2007)
			Epi R	TGGCTAATGGTTTGTCACCA			
	*S. capitis*	*sodA*	CT103F	TCAGATATTCAAACTGCAGTACG	103	58	In this study
			CT103R	CTACTTCACCTTTTTCTTCAGA			
	*S. saprophyticus*	*sodA*	SA52F	TGGACACTTAAACCACTCACTA	52	55	In this study
			SA52R	CTTCTGATTTGGAGTTAAT			

Species-specific PCR primers were evaluated in singleplex PCR mode, and in two groups (vide infra) of multiplex PCR reactions. Primer sets for multiplex PCR were divided based on the target fragment sizes into group 1 (*S. haemolyticus*, *S. pasteuri*, *S. warneri*, and *S. xylosus*) and group 2 (*S. capitis, S. caprae, S. epidermidis*, and *S. saprophyticus*). Group 1 PCR products comprised four *sodA* gene fragments; group 2 PCR products contained three *sodA* with one gseA gene fragments. The sizes of all PCR products were noticeably different.

The PCR mixtures contained 50 nM each primer and 0.1 μg of genomic DNA. The thermal cycling conditions were as follows: 1 cycle of 5 min at 95 °C, followed by 30 cycles of 30 s at 95 °C, 30 s at 55 °C (group 1) or 53 °C (group 2), and 30 s at 72 °C. The final step comprised 7 min at 72 °C. After PCR amplification, 5 μL of each reaction mixture was analyzed on a 2% agarose gel.

### Phylogenetic comparison

The phylogenetic relatedness of staphylococcal species was determined by sequence analysis of 16S rRNA and *sodA* genes. The 16S rRNA and *sodA* sequences were obtained from GenBank, and phylogenetic trees were constructed using ClustalW. A bootstrap analysis with 100 replicates was conducted to obtain confidence levels for the branches.

### Antimicrobial resistance

Antimicrobial resistance to 19 antibiotics was assessed using the disc diffusion method, in accordance with the standards of the Clinical and Laboratory Standards Institute (CLSI 2014). All isolates were incubated on Tryptic Soy Broth (TSB; Difco) medium at 37 °C, and the optical density (OD) at 600 nm of cultures was adjusted to 0.5 using a spectrophotometer. Mueller–Hinton agar (MHA; Oxoid, Hampshire, UK) was dispensed onto plastic culture plates to yield a uniform depth of 4 mm. A sterile swab was dipped into the OD-adjusted bacterial suspension and streaked onto the entire MHA surface. After streaking, the inoculum was dried and an antimicrobial disc was applied using a dispenser. The plate was incubated at 37 °C for 24 h. Nineteen antimicrobial discs containing the following antibiotics were tested: penicillin (10 U), oxacillin (1 µg), gentamycin (10 µg), amoxicillin/clavulanic acid (30 µg), tetracycline (30 µg), cephalothin (30 µg), imipenem (10 µg), ciprofloxacin (5 µg), erythromycin (15 µg), telithromycin (15 µg), clindamycin (2 µg), chloramphenicol (30 µg), trimethoprim/sulfamethoxazole (25 µg), nitrofurantoin (300 µg), quinupristin/dalfopristin (15 µg), linezolid (30 µg), vancomycin (30 µg), rifampicin (5 µg), and cefoxitin (30 µg). All of the applied antibiotic disc plates were incubated at 37 °C for 24 h. *S. aureus* ATCC 25923 was used as the control.

### Production of virulence factors

The presence of bacterial virulence factors was analyzed as follows. Hemolytic activity was determined on plates with a blood agar base with 5% (v/v) sheep blood at 37 °C for 24 h. The formation of hemolysis zones around the colonies indicated a positive result. Lipolytic activity was estimated by streaking the isolates onto Tween 20 agar (10 g of peptone, 5 g of NaCl, 0.1 g of CaCl_2_, 20 g of agar, and 1 mL of Tween 20 per liter) and incubating at 37 °C for 24 h. The formation of an opaque halo around the colonies indicated a positive result. Proteolytic activity was assessed by inoculating the isolates onto modified TSA medium containing 1% skim milk and incubating at 37 °C for 24 h. A positive result was indicated by the formation of a halo around the colonies. DNase activity was determined by inoculating the isolates onto DNase agar containing the methyl green indicator dye (Oxoid). The plates were incubated for 24 h at 37 °C and examined for evidence of DNA hydrolysis. A positive result was indicated by the formation of a clear zone around the colonies.

To determine their ability to form a biofilm, the staphylococcal isolates were transferred to TSB medium and incubated at 37 °C for 18 h. A 1:100 dilution of the cultures was transferred to a 96-well polystyrene plate (SPL, Gyeonggi-do, South Korea) and incubated at 37 °C for 18 h. Following the incubation, the supernatant was removed and the formed biofilms were carefully washed with physiological saline to remove planktonic bacteria. The biofilms were dried at 60 °C for 30 min and stained with 1% (w/v) crystal violet for 30 min. Unbound crystal violet was then removed with physiological saline until the control well became colorless. Bound crystal violet in each well was solubilized with 33% (v/v) glacial acetic acid, and sample absorbance was measured at OD 570 nm using a microplate reader (PerkinElmer, Waltham, MA, USA). The biofilm-forming ability was classified according to absorbance at OD 570 nm as weak (A570 <0.40), moderate (0.40 < A570 < 0.80), or strong (A570 >0.80).

The enzymatic experiments and biofilm assays were repeated twice, with three replicates per experiment.

## Results and discussion

### Improvement of CNS identification methods

To investigate the prevalence of pathogenic CNS on leaf vegetables, presumptive staphylococcal species were isolated on a selective medium, Baird-Parker agar. In total, 55 bacterial colonies were randomly selected and identified as follows.

First, the API Staph test was used to identify the isolated staphylococcal species. This commercially available phenotypic identification system is based on biochemical reactions, is simple to use, and provides rapid results. All isolates were identified as staphylococci: three were *S. aureus* and the remaining 52 belonged to nine CNS species (*S. capitis, S. caprae, S. epidermidis, S. haemolyticus, S. hominis, S. pasteuri, S. saprophyticus, S. warneri, and S. xylosus*) (Table 3). Based on the recommendation of the API software, 80% homology with the API ID (% ID) was chosen as the high-probability cutoff for positive identification (Park et al. 2011); however, the analysis of ca. 20% of the isolates (12/55) resulted in low-probability species identification (% ID <80).

**Table 3:** Comparison of phenotypic and genotypic identification results

Strain	16S rRNA	Species-specific PCR	API Staph	HV
S-37	*S. capitis*	*S. capitis*	*S. capitis*	63.30
S-182	*S. capitis*	*S. capitis*	*S. capitis*	99.10
S-185	*S. capitis*	*S. capitis*	*S. capitis*	96.00
S-72	*S. caprae*	*S. caprae*	*S. caprae*	65.50
S-176	*S. caprae*	*S. caprae*	*S. caprae*	97.00
S-4	*S. epidermidis*	*S. epidermidis*	*S. epidermidis*	89.30
S-5	*S. epidermidis*	*S. epidermidis*	*S. epidermidis*	97.90
S-9	*S. epidermidis*	*S. epidermidis*	*S. epidermidis*	99.40
S-10	*S. epidermidis*	*S. epidermidis*	*S. epidermidis*	99.40
S-11	*S. epidermidis*	*S. epidermidis*	*S. epidermidis*	99.40
S-12	*S. epidermidis*	*S. epidermidis*	*S. epidermidis*	99.40
S-13	*S. epidermidis*	*S. epidermidis*	*S. epidermidis*	99.40
S-62	*S. epidermidis*	*S. epidermidis*	*S. epidermidis*	97.90
S-65	*S. epidermidis*	*S. epidermidis*	*S. epidermidis*	94.30
S-88	*S. epidermidis*	*S. epidermidis*	*S. epidermidis*	88.10
S-104	*S. epidermidis*	*S. epidermidis*	*S. epidermidis*	99.40
S-105	*S. epidermidis*	*S. epidermidis*	*S. epidermidis*	86.20
S-115	*S. epidermidis*	*S. epidermidis*	*S. epidermidis*	94.60
S-118	*S. epidermidis*	*S. epidermidis*	*S. epidermidis*	94.30
S-180	*S. epidermidis*	*S. epidermidis*	*S. epidermidis*	97.30
S-181	*S. epidermidis*	*S. epidermidis*	*S. epidermidis*	97.30
S-183	*S. epidermidis*	*S. epidermidis*	*S. epidermidis*	94.30
SS-23	*S. epidermidis*	*S. epidermidis*	*S. epidermidis*	97.90
S-8	*S. haemolyticus*	*S. haemolyticus*	*S. haemolyticus*	99.00
S-122	*S. haemolyticus*	*S. haemolyticus*	*S. haemolyticus*	80.60
S-123	*S. haemolyticus*	*S. haemolyticus*	*S. haemolyticus*	92.40
S-124	*S. haemolyticus*	*S. haemolyticus*	*S. aureus*	42.20
S-166	*S. haemolyticus*	*S. haemolyticus*	*S. haemolyticus*	87.00
S-167	*S. haemolyticus*	*S. haemolyticus*	*S. haemolyticus*	87.00
SS-13	*S. haemolyticus*	*S. haemolyticus*	*S. haemolyticus*	99.60
S-34	*S. pasteuri*	*S. pasteuri*	*S. simulans*	72.60
S-35	*S. pasteuri*	*S. pasteuri*	*S. simulans*	95.40
SS-1	*S. pasteuri*	*S. pasteuri*	*S. warneri*	46.80
SS-32	*S. pasteuri*	*S. pasteuri*	*S. warneri*	46.80
S-68	*S. saprophyticus*	*S. saprophyticus*	*S. saprophyticus*	96.30
S-98	*S. saprophyticus*	*S. saprophyticus*	*S. xylosus*	99.90
S-38	*S. warneri*	*S. warneri*	*S. aureus*	97.80
S-39	*S. warneri*	*S. warneri*	*S. xylosus*	99.90
S-66	*S. warneri*	*S. warneri*	*S. aureus*	35.80
S-100	*S. warneri*	*S. warneri*	*S. xylosus*	71.30
S-142	*S. warneri*	*S. warneri*	*S. hominis*	40.40
S-163	*S. warneri*	*S. warneri*	*S. warneri*	37.50
S-173	*S. warneri*	*S. warneri*	*S. warneri*	89.90
S-174	*S. warneri*	*S. warneri*	*S. warneri*	55.80
S-208	*S. warneri*	*S. warneri*	*S. warneri*	55.80
SS-3	*S. warneri*	*S. warneri*	*S. warneri*	89.90
SS-6	*S. warneri*	*S. warneri*	*S. warneri*	89.90
S-169	*S. xylosus*	*S. xylosus*	*S. xylosus*	99.70
S-170	*S. xylosus*	*S. xylosus*	*S. xylosus*	99.70
S-171	*S. xylosus*	*S. xylosus*	*S. xylosus*	99.70
S-172	*S. xylosus*	*S. xylosus*	*S. xylosus*	99.70
S-179	*S. xylosus*	*S. xylosus*	*S. xylosus*	99.70
SS-17	*S. xylosus*	*S. xylosus*	*S. xylosus*	99.90
SS-19	*S. xylosus*	*S. xylosus*	*S. xylosus*	99.70
SS-20	*S. xylosus*	*S. xylosus*	*S. xylosus*	99.70

*HV* homology value in percent (%) of API Staph

In addition to the API kit, several phenotypic systems based on colony characteristics, antibiotyping patterns, saccharide utilization, and enzyme production are available for rapid and accurate identification of staphylococci (Aldea-Mansilla 2006). However, these diagnostic systems are considered to be a primary means for staphylococcal identification as their ability to provide reliable results is limited, mainly because of phenotypic differences between strains of the same species (Kooken et al. 2014).

Next, 16S rRNA sequencing was used to identify the bacterial isolates. All isolates were identified as CNS species, sharing 98–100% homology with the type strains; the sequences were deposited in GenBank under the accession numbers KX946134–KX946188. Homology values above 98% are considered as reliable; thus the identification results were credible. The isolates were identified as *S. capitis* (*n* = 3), *S. caprae* (*n* = 2), *S. epide*rmidis (*n* = 18), *S. haemolyticus* (*n* = 7), *S. pasteuri* (*n* = 4), *S. saprophyticus* (*n* = 2), *S. warneri* (*n* = 11), and *S. xylosus* (*n* = 8) (Table 3). The identification of 44 (80%) CNS isolates matched the identification by API Staph analysis. The ID values of four isolates from the remaining 11 (20%) isolates misidentified by the API kit were greater than 95.4%. These results show that, in terms of homology values, 16S rRNA analysis is superior to the API Staph test.

Although 16S rRNA analysis is a widely accepted method of bacterial species identification, it is unable to discriminate between closely related species. For example, in a previous study, *S. capitis* was misidentified as *S. epidermidis*, and *S. xylosus* was misidentified as either *S. cohnii* or *S. saprophyticus* (Ghebremedhin et al. 2008; Taponen et al. 2006). Accordingly, many kinds of highly conserved gene sequencing for CNS identification have been developed, including *hsp60*, *gap, sodA*, *tuf*, *rpoB*, and *dnaJ*; however, gene sequencing is time-consuming. The reliability of multiplex PCR targeting the conserved genes is same as that of gene sequencing, but the former method is simpler to employ and is more rapid (Blaiotta et al. 2004).

**Fig. 1:**
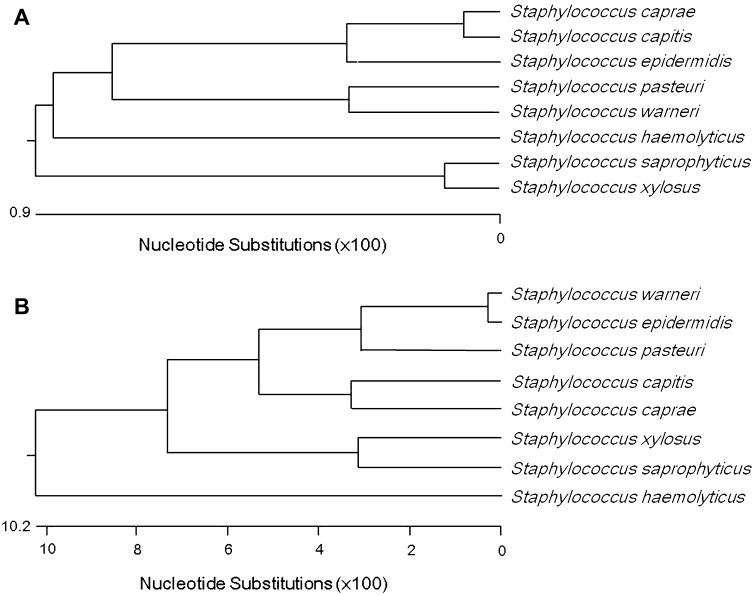
Phylogenetic tree construction using the Clustal W method based on 16S rRNA sequences (**a**), and *sodA* sequences of eight CNS species (**b**) obtained from GenBank. The value on each *branch* is the occurrence of the branching order in 100 bootstrapped trees. The *scale bar* represents 1% differences in nucleotide sequences

**Fig. 2:**
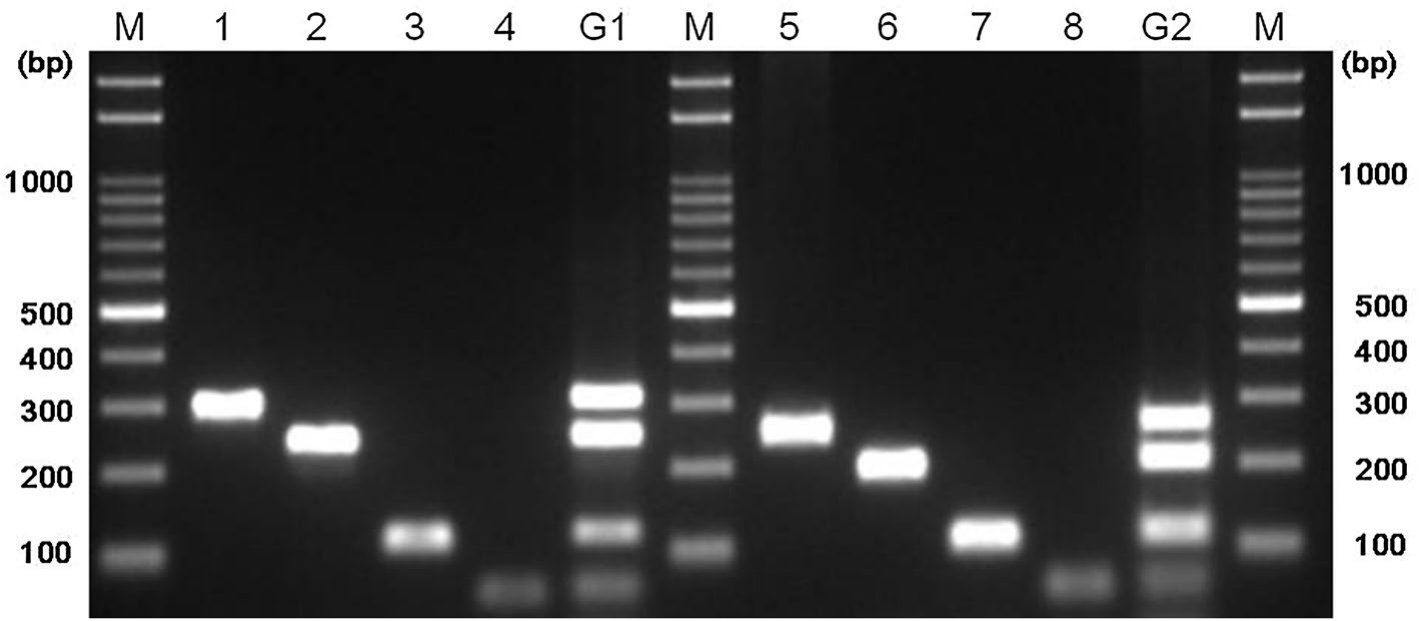
Agarose gel electrophoresis of PCR amplicons after amplification of species-specific singleplex and multiplex PCR targeting *sodA* gene from eight CNS species. *Lanes M* size marker; 1, *S. xylosus*; 2, *S. pasteuri*; 3, *S. warneri*; 4, *S. haemolyticus*, G1: group 1 (*S. xylosus, S. pasteuri, S. warneri,* and *S. haemolyticus*); 5, *S. caprae*; 6, *S. epidermidis*; 7, *S. capitis*; 8, *S. saprophyticus*, G2: group 2 (*S. caprae, S. epidermidis, S. capitis*, and *S. saprophyticus*)

We performed species-specific PCR using primers targeting the *sodA* gene, which encodes manganese-dependent superoxide dismutase, a key enzyme of oxygen defense systems (Fridovich 1995). In this study, seven new primer sets specifically targeting *sodA* from seven CNS species (*S. capitis*, *S. caprae*, *S. haemolyticus*, *S. pasteuri*, *S. saprophyticus*, *S. warneri*, and *S. xylosus*) were designed and evaluated (Table 2). Each primer set was designed to bind specifically to its target gene (Fig. S1), and not to those of other species (data not shown). *S. aureus* R0001 and the *Escherichia coli* DH5α were used as negative controls and were not detected by any PCR primer set. As shown in Table 3, all isolates were identified as CNS species, consistent with the results of 16S rRNA analysis. To compare the accuracy of 16S rRNA sequencing and *sodA*-targeting PCR, ClustalW phylogenetic trees were constructed with 16S rRNA and *sodA* sequences from eight type strains. The results indicated a higher divergence of the staphylococcal *sodA* gene than of 16S rRNA (Fig. 1). To increase the efficiency of detection, multiplex PCR assays were set up in two groups. In each multiplex PCR assay, a mixture of DNA from four CNS species yielded four PCR product bands of the expected sizes after gel electrophoresis (Fig. 2).

Previous studies have used *sodA* as a target for the identification of staphylococcal species (Iwase et al. 2007; Kooken et al. 2014; Poyart et al. 2001). However, in the majority of these studies, additional steps following PCR, such as sequencing and homology comparisons, were required (Coton et al. 2010). In some studies where *sodA* was used as the target gene for species-specific PCR, gel electrophoresis was also used for the identification of species, without any additional steps: *S. carnosus*, *S. simulans* (Blaiotta et al. 2005), *S. equorum* (Blaiotta et al. 2004), and *S. hyicus* (Voytenko et al. 2006). However, those studies were reported singleplex PCR method for CNS identification. In the current study, two groups of multiplex PCR primer sets that could discriminate eight CNS species were evaluated. Compared with conserved gene sequencing, species-specific multiplex PCR is more economical because it requires less time, with lower cost, without killing the reliability of identification. Furthermore, identification based on the *sodA* gene sequence can be extended to *Staphylococcus* species other than the seven CNS species evaluated in this study.

### Antimicrobial resistance of CNS

CNS are commonly found in food, environment, and clinical setting (Huber et al. 2011), and were recently recognized as etiologic agents of animal and human infectious diseases (Tremblay et al. 2014). Antimicrobial resistance is an important virulence factor (Wang et al. 2006), but little information is available on the prevalence of antimicrobial resistance in CNS from leaf vegetables. Antimicrobial resistance of CNS isolates was therefore examined in the current study. The distribution of resistance to 19 antimicrobial agents is presented in Table 4: 92.72% of the CNS isolates (51/55) showed resistance to at least one antimicrobial agent (Table 4); 83.64% of the CNS isolates (46/55) showed multidrug resistance. *S. haemolyticus* isolates were resistant to a lot more antibiotics than other CNS isolates. *S. haemolyticus*, the second most frequently isolated CNS from nosocomial infections, is resistant to multiple antibiotics (Brzychczy-Wloch et al. 2013). In the current study, resistance to penicillin, erythromycin, and oxacillin was frequently observed in the resistant isolates, but none of them were resistant to nitrofurantoin.

**Table 4:** Antimicrobial resistance and virulence enzyme production

Species	Strain	Antibiotics	Hemolysin	Lipase	Lecithinase	DNase	Protease	Biofilm formation^a^
*S. capitis*	S-37	E, CIP, DA	◯^b^	−^b^	−	◯	◯	+++
*S. capitis*	S-182	P, CIP, AMC	◯	−	−	◯	◯	+++
*S. capitis*	S-185	−	◯	−	−	◯	◯	+++
*S. caprae*	S-72	E	◯	−	◯	◯	◯	++
*S. caprae*	S-176	−	◯	−	◯	◯	◯	++
*S. epidermidis*	S-4	P, CN, E, OX, FOX, IPM	◯	◯	−	−	◯	++
*S. epidermidis*	S-5	P, CN, E, OX, FOX, TE, DA, VA	◯	◯	◯	−	◯	+
*S. epidermidis*	S-9	P	−	◯	−	◯	◯	+
*S. epidermidis*	S-10	P	−	◯	−	−	◯	+
*S. epidermidis*	S-11	P, QD	−	◯	−	−	◯	++
*S. epidermidis*	S-12	P, OX, LZD	−	◯	−	−	◯	++
*S. epidermidis*	S-13	P, CN, OX	◯	◯	−	−	◯	+
*S. epidermidis*	S-62	CIP	◯	◯	−	◯	◯	++
*S. epidermidis*	S-65	P, E	◯	◯	−	◯	◯	++
*S. epidermidis*	S-88	−	◯	−	−	◯	−	+
*S. epidermidis*	S-104	C, QD	−	◯	−	−	◯	++
*S. epidermidis*	S-105	P, OX, LZD	−	◯	−	−	◯	+
*S. epidermidis*	S-115	P, QD	◯	◯	−	−	◯	++
*S. epidermidis*	S-118	P, E, AMC	−	◯	−	−	◯	++
*S. epidermidis*	S-180	P, E, OX, FOX	◯	◯	−	−	◯	++
*S. epidermidis*	S-181	P, E, OX, FOX, IPM	◯	◯	◯	−	◯	++
*S. epidermidis*	S-183	CIP, DA	◯	◯	◯	−	◯	++
*S. epidermidis*	SS-23	P, CN, QD	◯	−	−	−	◯	++
*S. haemolyticus*	S-8	P, CN, E, CIP, TE, LZD	◯	−	−	−	−	++
*S. haemolyticus*	S-122	P, CN, C, RD	◯	−	−	−	◯	+++
*S. haemolyticus*	S-123	P, CN, E, OX, FOX, C, CIP	◯	−	−	◯	◯	++
*S. haemolyticus*	S-124	P, CN, E, OX, FOX, C, CIP, DA	◯	−	−	−	◯	+++
*S. haemolyticus*	S-166	P, CN, OX, FOX, C, CIP, DA	◯	−	−	−	−	+++
*S. haemolyticus*	S-167	P, OX, FOX, KF	◯	−	◯	◯	−	+++
*S. haemolyticus*	SS-13	P, CN, E, OX, FOX, C, CIP	◯	−	◯	◯	◯	++
*S. pasteuri*	S-34	P, E, C	◯	−	−	−	−	++
*S. pasteuri*	S-35	P, E	◯	−	−	−	−	++
*S. pasteuri*	SS-1	P, CN, E, AMC	◯	−	−	−	−	++
*S. pasteuri*	SS-32	P, CN, E	−	−	−	−	−	++
*S. saprophyticus*	S-68	E, OX, TE, RD	◯	−	−	−	−	+
*S. saprophyticus*	S-98	E, OX, FOX	−	−	−	−	−	++
*S. warneri*	S-38	P, CN, E, C, TE, KF	◯	◯	◯	−	◯	++
*S. warneri*	S-39	P, E, OX, FOX, CIP, KF	◯	◯	◯	−	−	++
*S. warneri*	S-66	P, CN, E, CIP, DA, KF	−	−	◯	−	−	++
*S. warneri*	S-100	−	◯	◯	◯	−	−	++
*S. warneri*	S-142	P, CN, OX, DA	◯	◯	−	−	◯	++
*S. warneri*	S-163	P, CN, VA	◯	◯	−	−	◯	+
*S. warneri*	S-173	P, CN, OX, FOX, AMC, KF	◯	−	◯	◯	−	++
*S. warneri*	S-174	P, CN, CIP	◯	◯	◯	−	◯	+
*S. warneri*	S-208	P, E, C, DA, AMC, TEL	◯	◯	◯	−	◯	++
*S. warneri*	SS-3	P, CN, E, C, AMC	◯	◯	−	−	−	++
*S. warneri*	SS-6	P, CN, E, C, TE, KF	◯	◯	◯	−	−	+
*S. xylosus*	S-169	P, E, OX	−	−	−	◯	−	+++
*S. xylosus*	S-170	P, E, OX, FOX, RD	◯	−	◯	◯	◯	+++
*S. xylosus*	S-171	P, SXT	−	◯	◯	−	◯	++
*S. xylosus*	S-172	P, OX	−	◯	◯	−	◯	++
*S. xylosus*	S-179	P, OX	−	−	◯	−	−	++
*S. xylosus*	SS-17	C, RD	−	◯	◯	◯	−	++
*S. xylosus*	SS-19	P	−	◯	−	−	◯	++
*S. xylosus*	SS-20	P, OX	−	◯	−	−	◯	++

^a^ Biofilm formation capacity: +, weak (A570 <0.40); ++, moderate (0.40 < A570 < 0.80); +++, strong (A570 >0.80)

^b^ Enzyme activity: ◯, positive; −, negative

### CNS virulence factors

Pathogenicity of staphylococci is linked to the production of virulence-associated enzymes that are responsible for the development of disease (Taponen and Pyorala 2009). Various virulence factors have been identified in *S. aureus* strains from diverse sources (Kim et al. 2015); however, little is known about the virulence factors produced by CNS. The CNS strains isolated in the current study produced diverse virulence-associated enzymes, including hemolysin (69.09%), protease (65.45%), lipase (54.54%), lecithinase (36.36%), and DNase (29.09%) (Table 4). Notably, all species produced hemolysin. Previous studies revealed that staphylococcal hemolysin plays a specific role in the pathogenesis of various infectious diseases, such as neurotoxia and peritonitis. The consumption of fresh vegetables contaminated with CNS may therefore lead to human illness (Dahlberg et al. 2015).

In addition to virulence-associated enzymes, it has been proposed that CNS biofilms are an important cause of recurrent and chronic infectious diseases in animals (Oliveira et al. 2006). Biofilm-associated bacteria are highly adhesive and exhibit decreased susceptibility to detergents, biocides, and antimicrobial agents (Donlan 2001). The biofilms therefore significantly increase the ability of bacteria to dwell in tissues and on inanimate surfaces. In the current study, biofilm formation by the CNS isolates was investigated by crystal violet staining. Based on this assay, the biofilm-forming ability of the CNS isolates was classified as weak, moderate, or strong. From the 55 isolates, the biofilm-forming ability of 9 (16.36%), 36 (65.45%), and 10 (18.18%) isolates was classified as weak, moderate, and strong, respectively (Table 4). On the species level, the biofilm-forming ability was most pronounced in *S. haemolyticus* and *S. xylosus*. *S. haemolyticus* can cause serious infection in humans, leading to endocarditis, urinary tract infections, and septicemia (Piette and Verschraegen 2009). Although *S. xylosus* is not commonly associated with human infection, it has been isolated in some cases of endocarditis, pyelonephritis, and septicemia (Gozalo et al. 2010). The presence of multidrug-resistant CNS with biofilm-forming ability on fresh vegetables is concerning. Because some CNS species are more resistant to antibiotics than other species, their identification on a species level is important for the control of CNS infections.

## Conclusion

We designed *sodA* primers specific to seven CNS species for rapid CNS identification, and confirmed. Using these primers, 55 staphylococcal isolates from leaf vegetables were successfully identified. These primers are also suited to multiplex PCR. The new method has the simplicity of phenotypic identification methods with the accuracy of genotypic identification. This method may also be combined with real-time PCR and DNA chip technology to quantify *sodA* expression in specific *Staphylococcus* species. Rapid and accurate CNS identification would allow tracking of the transmission of pathogenic CNS and contribute to the control of antibiotic-resistant CNS.

## Electronic supplementary material

Below is the link to the electronic supplementary material.

Supplementary material 1 (DOCX 132 kb)
