# Supplementary material for: Improved multiplex PCR primers for rapid identification of coagulase-negative staphylococci
Source: Arch Microbiol. 2017 Aug 9;200(1):73–83. doi: 10.1007/s00203-017-1415-9 (PMC5758691; doi:10.1007/s00203-017-1415-9)
Supplement: Supplementary file 1 — Supplementary material 1 (DOCX 132 kb) [file 203_2017_1415_MOESM1_ESM.docx]

Supplementary Figure


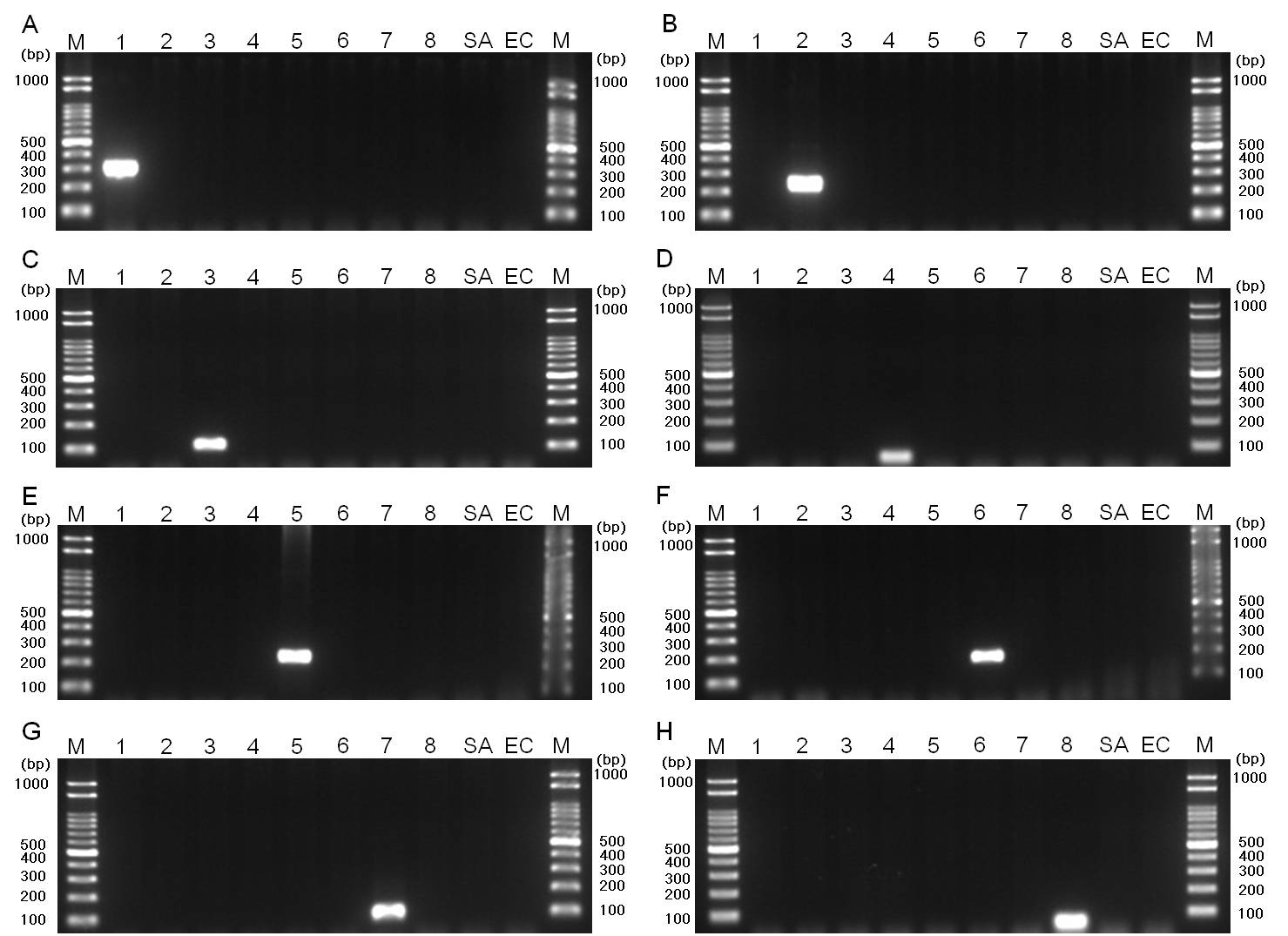


Figure S1. Agarose gel electrophoresis of PCR amplicons after amplification of singleplex PCR using species-specific primers (A: SX297, B: PA237, C: SW110, D: HA54, E: CR252, F: Epi, G: CT103, and H: SA52) targeting *sodA* gene from eight CNS species. Lanes M: Size marker; 1: *S*. *xylosus*, 2: *S*. *pasteuri*, 3: *S*. *warneri*, 4: *S*. *haemolyticus*, 5: *S*. *caprae*, 6: *S*. *epidermidis*, 7: *S*. *capitis*, 8: *S*. *saprophyticus*; SA and EC: negative control of each species-specific singleplex PCR reactions, SA: *Staphylococcus aureus* R0001, EC: *Escherichia coli* DH5α
